# Diagnosis of Acute Central Dizziness With Simple Clinical Information Using Machine Learning

**DOI:** 10.3389/fneur.2021.691057

**Published:** 2021-07-12

**Authors:** Bum Joon Kim, Su-Kyeong Jang, Yong-Hwan Kim, Eun-Jae Lee, Jun Young Chang, Sun U. Kwon, Jong S. Kim, Dong-Wha Kang

**Affiliations:** ^1^Department of Neurology, Asan Medical Center, University of Ulsan, College of Medicine, Seoul, South Korea; ^2^Asan Medical Center, Asan Institute for Life Sciences, Seoul, South Korea; ^3^Nunaps Inc., Seoul, South Korea

**Keywords:** dizziness, vertigo, machine learning, stroke, vertebrobasilar insufficiency

## Abstract

**Background:** Acute dizziness is a common symptom among patients visiting emergency medical centers. Extensive neurological examinations aimed at delineating the cause of dizziness often require experience and specialized training. We tried to diagnose central dizziness by machine learning using only basic clinical information.

**Methods:** Patients were enrolled who had visited an emergency medical center with acute dizziness and underwent diffusion-weighted imaging. The enrolled patients were dichotomized as either having central (with a corresponding central lesion) or non-central dizziness. We obtained patient demographics, risk factors, vital signs, and presentation (non-whirling type dizziness or vertigo). Various machine learning algorithms were used to predict central dizziness. The area under the receiver operating characteristic curve (AUROC) was measured to evaluate diagnostic accuracy. The SHapley Additive exPlanations (SHAP) value was used to explain the importance of each factor.

**Results:** Of the 4,481 visits, 414 (9.2%) were determined as central dizziness. Central dizziness patients were more often older and male and had more risk factors and higher systolic blood pressure. They also presented more frequently with non-whirling type dizziness (79 vs. 54.4%) than non-central dizziness. Catboost model showed the highest AUROC (0.738) with a 94.4% sensitivity and 31.9% specificity in the test set (*n* = 1,317). The SHAP value was highest for previous stroke presence (mean; 0.74), followed by male (0.33), presentation as non-whirling type dizziness (0.30), and age (0.25).

**Conclusions:** Machine learning is feasible for classifying central dizziness using demographics, risk factors, vital signs, and clinical dizziness presentation, which are obtainable at the triage.

## Introduction

Acute dizziness and vertigo are common symptoms presented by patients admitted to emergency medical centers (EMCs) (1). Though dizziness is usually attributable to benign etiologies originating from peripheral causes, 5% of acute dizziness may be caused by cerebrovascular issues (2). Acute dizziness and vertigo are the most common presenting symptoms of vertebra-basilar ischemia (3), which shows a stepwise deterioration of poor prognosis when the diagnosis is inappropriately delayed (4).

Unfortunately, because there is no standard test or biomarker that can be used for the confirmatory diagnosis of central dizziness, verification of the etiology remains challenging. Many efforts have been made to distinguish central dizziness from peripheral dizziness, especially those utilizing extensive neurological examinations (5). However, to some extent, misdiagnosis stems from an overreliance on negative neurological examinations (2). Approximately 11% of medial posterior-inferior cerebellar artery infarction patients were shown to present with isolated vertigo, abnormal ocular manifestations, and imbalance (6). However, interpretation of the oculomotor findings often requires further examinations with experienced and specialized neuro-ophthalmology staff. While acute dizziness and vertigo are very commonly observed clinical symptoms, which most physicians, not only specialists, may encounter daily, misdiagnosis can lead to devastating results (7).

Therefore, clinicians require a simple and widely applicable method with high sensitivity that can significantly reduce misdiagnosis of central dizziness. Machine learning (ML) has previously been used and has shown an acceptable performance in predicting the characteristics and prognosis of ischemic stroke (8–11). Several studies have shown that ML can be used to analyze nysagmogram or postulography videos to diagnose the causes of dizziness, which still needs equipment to measure the nystagmus or posture (12, 13). Here, we used ML techniques to diagnose isolated acute dizziness patients visiting EMCs. As such, we used only simple clinical information to delineate the central causes of dizziness from peripheral causes. Additionally, we aimed to examine the feature importance of the ML model and understand its behavior.

## Materials and Methods

### Participants

Patients visiting the EMC of the Asan Medical Center with acute dizziness or vertigo were consecutively checked with diffusion-weighted imaging (DWI) to exclude central dizziness. In the present study, we have retrospectively recruited patients who visited the EMC presenting with acute dizziness or vertigo between January 2010 and December 2013 and received DWI before being discharged from the EMC.

We excluded patients refusing DWI or with contraindications for magnetic resonance imaging (MRI; i.e., pacemaker). In addition, patients were excluded who presented with symptoms indicative of nausea, even though the chief complaint was dizziness or vertigo, and were diagnosed with gastrointestinal disorders. Patients who presented with non-specific dizziness and were diagnosed with general weakness due to poor medical conditions, such as systemic infection or cancer, were also excluded from the final analysis. The Institutional Review Board of the Asan Medical Center approved this study. Informed consent was waived because of the retrospective design.

### Classification of the Cause of Dizziness

The cause of acute dizziness and vertigo was categorized based on the final diagnosis upon discharge from the EMC. The final diagnosis was based on extensive evaluation with neuroimaging and comprehensive evaluation by neurologists, otorhinolaryngologists, and emergency medicine physicians. All patients included received DWI. Additional neuroimaging procedures were performed for patients who were suspicious of vertebrobasilar insufficiency after consultation to the neurologists. Computed tomography angiography (CTA) or magnetic resonance angiography (MRA) were performed based on the clinician's decision.

The cause of dizziness was initially categorized as one of the following: (1) central, (2) peripheral, (3) psychogenic, (4) cardio-circulatory, and (5) non-specific. A diagnosis of central dizziness was dependent upon the identification of a focal structural lesion from the corresponding area. Central dizziness included patients with DWI lesions presenting acute ischemic stroke or a significant stenosis (more than 50%) at the vertebrobasilar system. Peripheral dizziness included patients diagnosed as benign paroxysmal positional vertigo, Meniere's disease, vestibule-neuritis, and other vestibulopathies. Patients diagnosed with depression, anxiety disorder, or hyperventilation were categorized as psychogenic dizziness. Cardio-circulatory dizziness included patients diagnosed as syncope or presyncope due to cardiac problems, such as symptomatic arrhythmias, causing insufficient cerebral circulation. Dizziness with an unclear etiology but excluded from being categorized as central causing dizziness by neuroimaging was determined to be non-specific dizziness. All causes of dizziness, except central dizziness, were regarded as non-central dizziness.

### Development and Evaluation of Model

Predictors included demographics (e.g., age and sex), previous medical history (e.g., history of hypertension, diabetes, hyperlipidemia, stroke, or coronary artery disease), systolic and diastolic blood pressure (BP), and heart rate. In the current study, we built classification models using various ML algorithms, including the radial basis function kernel support vector machine (SVM), random forest (RF), Catboost, and conventional logistic regression (LR).

The data was split by order of admission date (i.e., temporal validation) into a training set and a test set. Within a training set, multiple hyperparameters were tuned using a five-fold cross-validation. The loss function was negative a log-likelihood with class weights. Area under the curve of the receiver operating characteristic curve (AUROC) was measured to validate performance in a test set. Sensitivity and specificity were also calculated on a test set using threshold at which sensitivity on the training set was 99% or 99.9%, since not missing patients with central dizziness is a more critical factor not missing those with non-central dizziness.

In addition, to understand the reasoning behind certain ML model predictions, we used a Tree Explainer of SHapley Additive exPlanations (SHAP) value (https://github.com/skjang54/Asan_Central-Dizziness-and-Machine-Learning/) (14). The SHAP value (log-odds unit) identifies the degree of impact a predictor has on a prediction. A positive SHAP indicates that the feature drives an increase in the probability of central dizziness (response variable), and a negative SHAP implies that the feature decreases the probability. This approach provides local explanations by illustrating the attribution of a feature within a single patient. Also, the mean of the absolute values of SHAP explains the importance of each feature across the population (global explanation). The SHAP values of Catboost and RF models were computed by Tree Explainer, with SVM as the Kernel Explainer and LR as the Linear Explainer (SHAP version 0.34.0).

## Results

**Figure 1:**
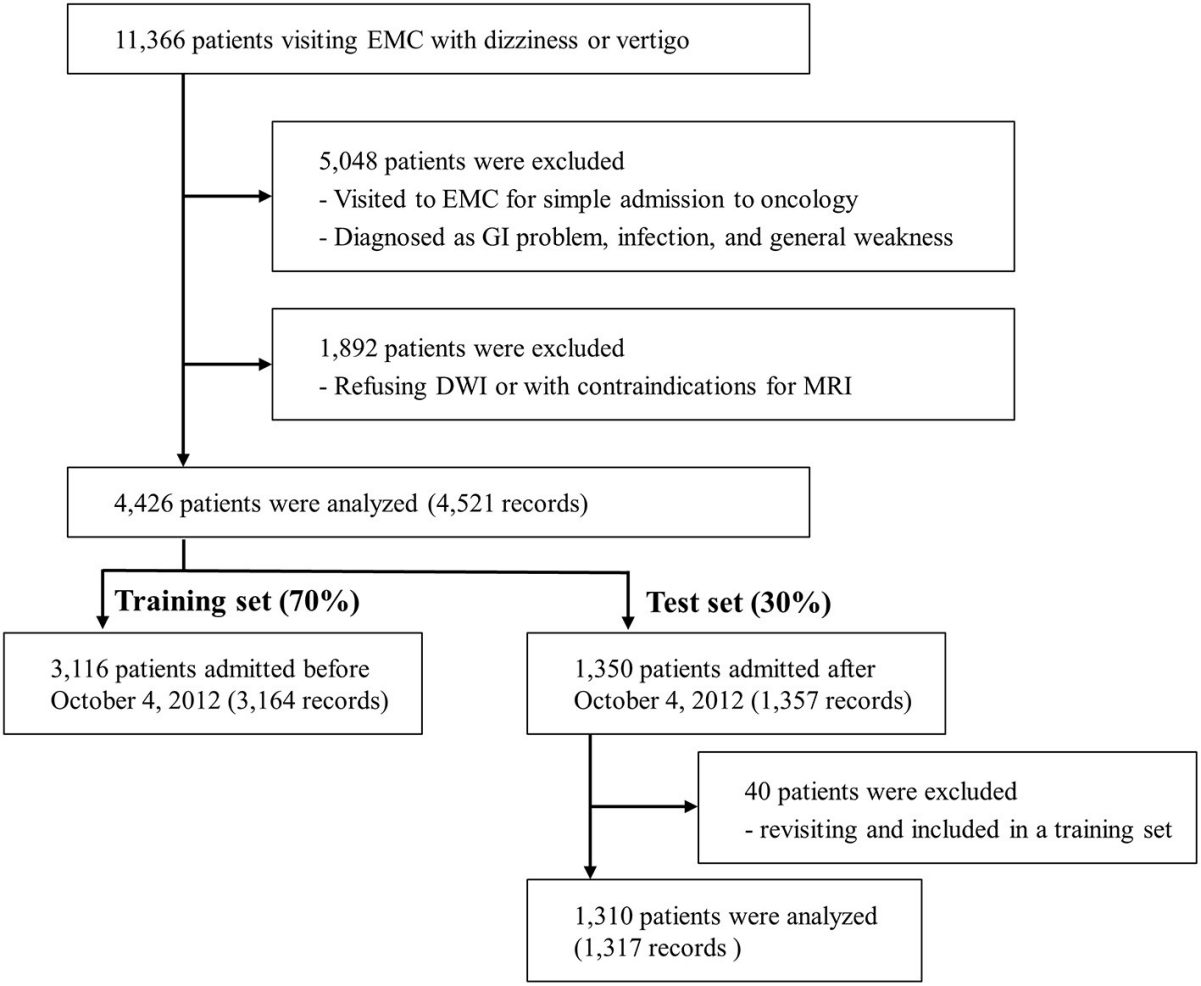
Flow diagram. EMC, emergency medical center; GI, gastrointestinal; DWI, Diffusion-weighted imaging; MRI, Magnetic resonance imaging.

Of the 11,366 patients who visited the EMC with dizziness or vertigo, 4,426 patients were included in the final analysis (Figure 1). All patients had DWI data and 989 patients had additional angiographic evaluation (CTA, *n* = 81 and MRA, *n* = 908). Of these final patients, 3,116 patients were included in the training set, and the remaining 1,310 patients were included in the test set. Within the period of the training set, 46 patients visited twice, and one patient visited three times. Only the first visit was used for the test set. The ratio of central to non-central causes of dizziness was not significantly different in the training set than the test set (Supplementary Table 1). Among 4,481 records, 414 (9.2%) were diagnosed with central dizziness. There was no missing data among clinical variables.

### Characteristics of Patients With Central Dizziness

As shown in Table 1, patients with central dizziness were older (66.1 ± 11.8 vs. 61.3 ± 11.5 years old; *p* < 0.001) and more often male than those with non-central dizziness (59.2 vs. 39.1%; *p* < 0.001). Patients with central dizziness showed a higher prevalence of hypertension (47.8 vs. 37.6%; *p* < 0.001), diabetes (24.2 vs. 13.1%; *p* < 0.001), current smoking (9.9 vs. 6.5%; *p* = 0.012), previous coronary artery disease (18.6 vs. 12.2%; *p* < 0.001), and history of stroke (24.2 vs. 7.7%; *p* < 0.001). Systolic BP was higher in patients with central dizziness than in those with non-central dizziness (148.3 ± 23.6 vs. 145.7 ± 22.0 mmHg; *p* = 0.032). The clinical presentation of non-whirling-type dizziness was observed more often in patients with central dizziness than in those with non-central dizziness (79.0 vs. 54.4%; *p* < 0.001).

**Table 1:** Baseline characteristics of the records of the enrolled patients with central and non-central dizziness.

	**Central dizziness^†^ (*n* = 414)**	**Non-central dizziness^†^ (*n* = 4,067)**	***p*-value^‡^**
Age (y)	66.1 ± 11.8	61.3 ± 11.5	<0.001
Sex (male)	245 (59.2)	1592 (39.1)	<0.001
Hypertension	198 (47.8)	1531 (37.6)	<0.001
Diabetes	100 (24.2)	531 (13.1)	<0.001
Hyperlipidemia	133 (32.1)	1,368 (33.6)	0.571
Current smoking	41 (9.9)	265 (6.5)	0.012
History of previous coronary artery disease	77 (18.6)	495 (12.2)	<0.001
History of previous stroke	100 (24.2)	313 (7.7)	<0.001
Systolic blood pressure (mmHg)	148.3 ± 23.6	145.7 ± 22.0	0.032
Diastolic blood pressure (mmHg)	87.7 ± 15.8	88.1 ± 14.0	0.584
Heart rate (beat/min)	75.2 ± 15.1	74.6 ± 14.0	0.425
Presentation of dizziness			<0.001
Non-whirling type dizziness	327 (79.0)	2,211 (54.4)	
Vertigo	87 (21.0)	1,856 (45.6)	

† *Values represented as frequency (percentage) or mean ± SD*.

‡ *P-values were calculated using t-test for continuous variables and χ^2^-test for categorical variables*.

### Machine Learning Predicting Central Dizziness

**Figure 2:**
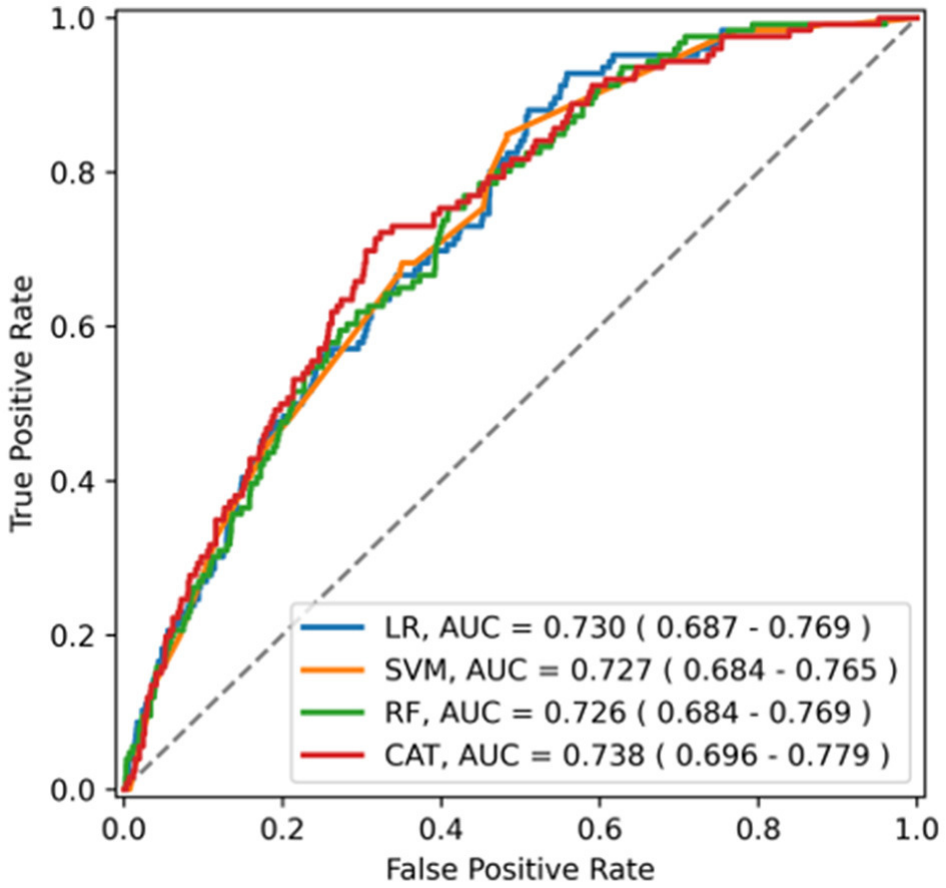
The receiver operating characteristics curve of the model for central dizziness. LR, logistic regression; SVM, support vector machine; RF, random forest; CAT, Catboost; AUC, area under the receiver operating characteristic curve.

The ability of the ML algorithms to discriminate between central and non-central dizziness is shown in Figure 2. In the ROC analysis, the models achieved an AUROC of 0.730 (0.690–0.771) in LR, 0.727 (0.687–0.767) in SVM, 0.726 (0.686–0.768) in RF, and 0.738 (0.693–0.780) in Catboost, suggesting moderate predictive accuracy with highest performance by Catboost but without statistically significant difference among models. (Catboost vs. LR: *p* = 0.443; Catboost vs. SVM: *p* = 0.263; Catboost vs. RF: *p* = 0.099). Sensitivity and specificity were also similar among the models, evaluated in the independent test set (Table 2). At a classification threshold of 99% sensitivity in the training set, the models showed a sensitivity of 97.6–99.2% and a specificity of 10.5–18.6%. At a threshold of 99.9% sensitivity, the models showed a sensitivity of 99.2–100% and a specificity of 4.6–8.1%.

**Table 2:** Sensitivity and specificity of the classification models in the independent test set.

	**Sensitivity**	**Specificity**
**A. Threshold at which sensitivity is 99% in training set**		
Logistic regression	0.992 (0.976–1.000)	0.107 (0.088–0.125)
Support vector machine	0.984 (0.960–1.000)	0.157 (0.136–0.177)
Random forest	0.992 (0.976–1.000)	0.125 (0.108–0.144)
Catboost	0.976 (0.944–1.000)	0.167 (0.146–0.190)
**B. Threshold at which sensitivity is 99.9% in training set**		
Logistic regression	0.992 (0.968–1.000)	0.068 (0.055–0.083)
Support vector machine	0.992 (0.976–1.000)	0.116 (0.099–0.134)
Random forest	0.992 (0.976–1.000)	0.060 (0.046–0.073)
Catboost	1.000 (1.000–1.000)	0.046 (0.034–0.060)

### Factors Predicting Central Dizziness

**Figure 3:**
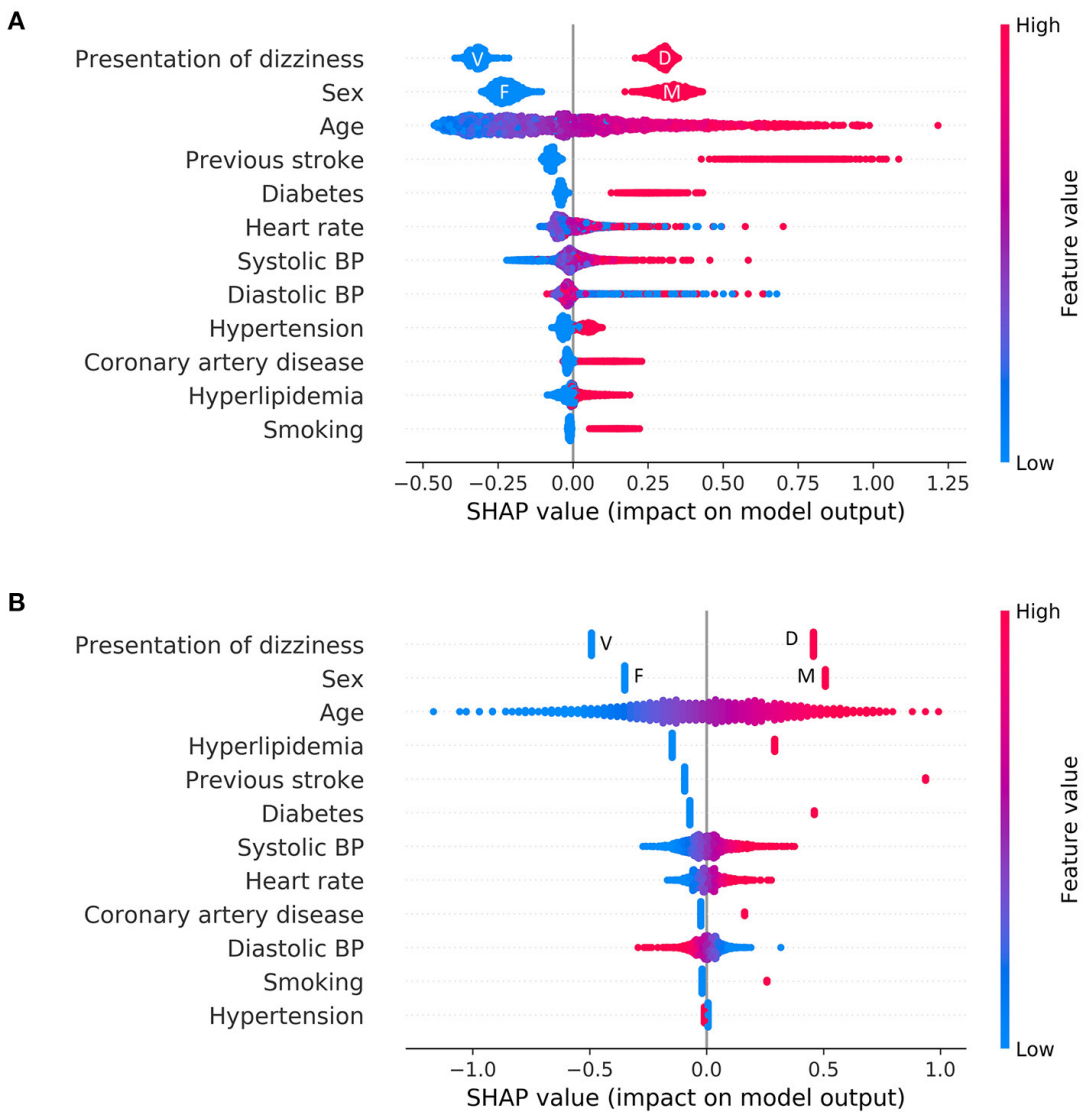
Feature attributions of all features. Summary plot of SHAP values for all features: **(A)** Logistic regression **(B)** Catboost. Each point represents the feature attribution on the log-odds scale for one patient in the training set. Continuous variables were colored by feature value. In the case of categorical variables, except for representation of dizziness and sex, the red color denotes a positive (i.e., a patient has the feature), and the blue color denotes negative. F, female; M, male; V, vertigo; D, dizziness.

Overall feature attributions of the Catboost model were compared with those of the LR as shown in Figure 3. The mean Catboost SHAP value was highest for the presence of a previous stroke history (0.74 ± 0.12), followed by male (0.33 ± 0.04), presentation as non-whirling-type dizziness (0.30 ± 0.02), and age (0.25 ± 0.18). The mean of the absolute values of SHAP was highest for the presentation of dizziness (0.31 ± 0.02), followed by sex (0.27 ± 0.06), age (0.25 ± 0.18), and history of previous stroke (0.14 ± 0.20) in Catboost (Figure 3A). These four features—presentation of non-whirling-type dizziness, sex, and age—were also factors with relatively strong impacts on other algorithms, including LR (Figure 3B and Supplementary Figure 1).

**Figure 4:**
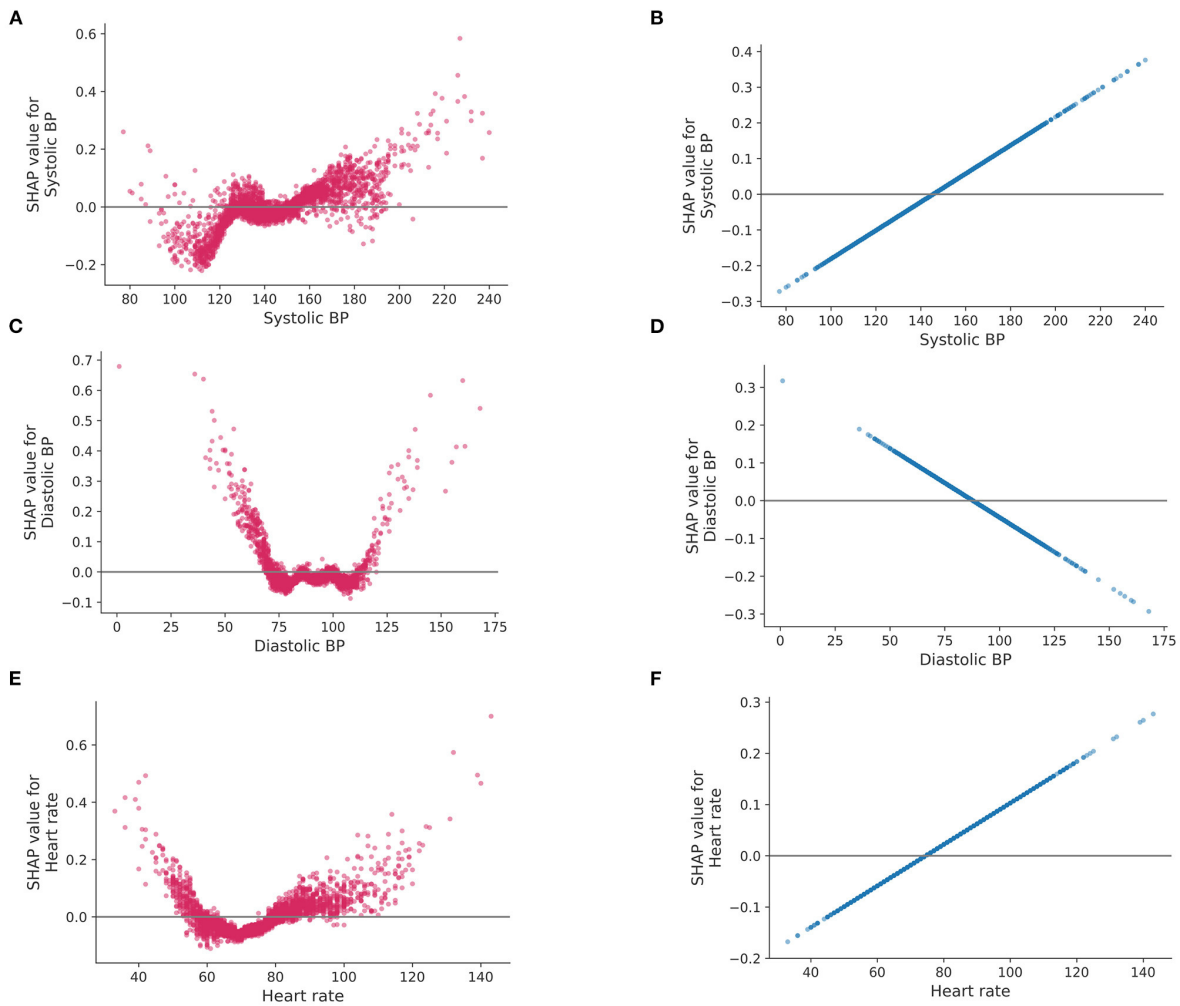
SHAP values for a single feature. SHAP values (impact of each feature on the model) of logistic regression and Catboost are represented on the left and right side, respectively. Systolic blood pressure **(A,B)**, diastolic blood pressure **(C,D)** and heart rate **(E,F)**. BP, blood pressure.

The difference in the way to use features between LR and Catboost can be seen in Figure 4. The plots represent how a single feature affects the classifier according to their values. As shown in Figure 4, lower than normal systolic BP contributed to a greater negative prediction for central dizziness, whereas higher BP, between 125 and 150 mmHg, showed a neutral SHAP value. Systolic BP over 150 mmHg showed a positive SHAP value, indicating the model considers patients having the range of systolic BP at high risk for central dizziness (Figure 4A). Additionally, diastolic BP under 75 or higher than 125 mmHg increased the risk for central dizziness (Figure 4C; U-shape curve). Similarly, heart rates lower than 60 and higher than 80 bpm showed positive SHAP values (Figure 4E). However, the LR model considered that as the value of feature increases [systolic BP (B), and heart rate (D)] or decreases [diastolic BP (F)], the feature contributes to increasing the log-odds of central dizziness linearly. The RF seemed to have a similar pattern as Catboost, and the SVM appeared to have a smooth curve because of the radial basis function kernel (Supplementary Figure 2).

## Discussion

In the current study, 9% of patients visiting the EMC showed central dizziness. The ML algorithms designed to predict central dizziness using simple clinical data obtained from triage showed moderate predictive accuracy. The presentation type of dizziness (non-whirling-type dizziness), age (older), sex (male), and history of stroke (present) were shown to be important factors for predicting central dizziness in the Catboost model.

Previous studies have differentiated central dizziness from peripheral causes of vertigo by extensive neuro-ophthalmological examination, including the head-thrust test, gaze-evoked nystagmus, and skew deviation (15). These three tests were proven to be even more sensitive than neuroimaging (15). However, these tests are typically difficult to apply for non-neuro-otology specialists. Alternatively, many scoring systems have been applied to diagnose central dizziness. The modified ABCD2 (age, BP, clinical presentation, and diabetes) score showed an AUROC curve of 0.79 (16). Another scoring system that used eight items, the TriAGE+ score, had an AUROC of 0.82 (17). However, these scoring systems are complicated and still require neurological tests, such as testing for cerebellar dysfunction or focal weakness. Among patients without weakness, the AUROC of the ABCD2 score was lowered to 0.63 (17). Considering that ~10% of patients with cerebellar infarction present with isolated vertigo (6), it may be inappropriate to differentiate central dizziness based on these scoring systems. Our ML-based diagnosis of central vertigo based specifically on simple clinical information, in the absence of neurological information, showed strong predictive power in classifying central dizziness reaching accuracies close to previous scoring systems.

ML has recently shown promising results in various medical fields, some of which have been validated in real-world settings (18). In ML, algorithms are designed to identify important features and/or complicated relationships between these features in an attempt to predict or classify response variables; this is in contrast to rule-based algorithms that use features defined manually. In the current study, Catboost had the highest AUROC among the ML models (0.738) and had 16.7% of specificity at 97.6% of sensitivity and 4.6% of specificity at 100% of sensitivity. These results indicate that, if the model is used to differentiate between central dichotomously in practice, 2.4% of patients with central dizziness would be misdiagnosed, while 16.7% of those with non-central dizziness do not require further neuroimaging services.

We further uncovered important predictors of central dizziness and, moreover, how these variables impact the decision making of the Catboost model using SHAP. Based on our results, previous stroke history, sex (males), presentation of dizziness, and age (older) were the most important factors for classifying central dizziness (Supplementary Figure 1). However, according to SHAP, other factors, such as heart rate and systolic and diastolic BP, were still proven to be useful for classifying central dizziness. However, these factors displayed some differences, with higher systolic and diastolic BP showing positive SHAP values. However, lower systolic BP was correlated with lower SHAP values, whereas lower diastolic BP was associated with higher SHAP values. Heart rate results were similar to that of diastolic BP. Patients with low or high heart rates may have a change of subclinical arrhythmias that may increase the risk of central dizziness. SHAP value patterns exhibit non-linear characteristics using the Catboost model, which is similar to previously observed effects of blood pressure or heart rate on stroke risk. Meanwhile, a linear model, such as LR, showed linearly increased or decreased SHAP values as the variables increase (Figure 4).

There are some noteworthy limitations to this study. First, this was a single-center study and may have a limitation in generalizability. Though we included a large number of patients, consecutively to reduce the bias, external validation may be needed to strengthen our results. Second, the clinical information was based on a conservative format that evaluated dizziness based on the presentation of dizziness and risk factors (19). A more updated algorithm that delineates dizziness using timing and triggers may show different results. However, using the presentation of dizziness and risk factors is still a widely accepted clinical approach for differentiating central dizziness. Finally, since ML has an advantage in processing large data, combining findings with video nystagmography or video oculography may enhance the predictive power of the algorithms. However, here we simply used clinical information for the ML input in an attempt to diagnose central dizziness, a strategy that may be more applicable for the non-neuro-otology specialists. Furthermore, as we only used simple data obtainable from the triage, there was no missing clinical data throughout the study.

Our results show that ML models for predicting central dizziness are feasible and require only simple clinical data and the presentation of dizziness. This tool for diagnosing central dizziness may be extremely helpful for non-neuro-otology specialists in determining the priorities of urgent patients and differentiating central dizziness from non-central dizziness in clinical practice.

## Data Availability Statement

The raw data supporting the conclusions of this article will be made available by the authors, without undue reservation.

## Ethics Statement

The studies involving human participants were reviewed and approved by Institutional Review Board of the Asan Medical Center. Written informed consent for participation was not required for this study in accordance with the national legislation and the institutional requirements.

## Author Contributions

BK contributed by the conceptualization, data curation, and writing the draft preparation. S-KJ contributed by data curation, formal analysis, and draft preparation. Y-HK contributed by formal analysis, visualization, and critical revision of the manuscript. E-JL contributed by conceptualization and critical revision of the manuscript. JC contributed by data curation and revision of the manuscript. SK and JK contributed by supervision and critical revision of the manuscript. D-WK contributed by the conceptualization, supervision, and writing the draft preparation and critical revision of the manuscript. All authors contributed to the article and approved the submitted version.

## Conflict of Interest

Y-HK was employed by company Nunaps Inc. The remaining authors declare that the research was conducted in the absence of any commercial or financial relationships that could be construed as a potential conflict of interest.
