# Supplementary material for: Diagnosis of Acute Central Dizziness With Simple Clinical Information Using Machine Learning
Source: Front Neurol. 2021 Jul 12;12:691057. doi: 10.3389/fneur.2021.691057 (PMC8313110; doi:10.3389/fneur.2021.691057)

이 문서는 서울아산병원의 문서입니다. 동의없이 수정, 변경 및 복사할 수 없습니다.

**SUPPLEMENTAL MATERIAL**

# Diagnosis of Acute Central Dizziness with Simple Clinical Information using Machine Learning Techniques

Bum Joon Kim[a, *], Su-Kyeong Jang[a, *], Yong-Hwan Kim[b], Eun Jae Lee[a], June Young Chang[a], Sun U. Kwon[a], Jong S. Kim[a], Dong-Wha Kang[a]

[a]Departments of Neurology, Asan Medical Center, Seoul, Korea

[b]Asan Institute for Life Sciences, Asan Medical Center, Seoul, Korea

* Bum Joon Kim and Su-Kyeong Jang equally contributed to this paper.

서울아산병원 Asan Medical Center

ASAN FOUNDATION 아산재단

이 문서는 서울아산병원의 문서입니다. 동의없이 수정, 변경 및 복사할 수 없습니다.

**Supplemental Tables List**

**Supplementary Tables**

**Supplementary Table 1.** Baseline characteristics of a training set and test set

**Supplementary Table 2.** Coefficients of logistic regression

**Supplementary Figure Legends**

**Supplementary Figure 1.** Relative importance of each feature

Ratio of the mean of the absolute value of SHAP for each feature to the sum of them. Features are listed in order of feature importance in logistic regression.

**Supplementary Figure 2.** Feature attributions for Support vector machine and Random forest.

SHAP values as a function of age (first row), systolic (second row) and diastolic blood pressure (third row), and heart rate (fourth row) for Support vector machine (left, yellow) and random forest (right, green).

서울아산병원 Asan Medical Center ASAN FOUNDATION 아산재단

이 문서는 서울아산병원의 문서입니다. 동의없이 수정, 변경 및 복사할 수 없습니다.

**Supplementary Table 1.** Baseline characteristics of a training set and a test set

| | Training set† (n = 3,164) | Test set† (n = 1,317) | *P*-value‡ |
|---|---|---|---|
| Cause of dizziness | | | 0.665 |
| Central | 288 (9.1) | 126 (9.6) | |
| Non-central | 2876 (90.9) | 1191 (90.4) | |
| Presentation | | | <0.001 |
| Dizziness | 1641 (51.9) | 897 (68.1) | |
| Vertigo | 1523 (48.1) | 420 (31.9) | |
| Age (y) | 61.6 ±11.5 | 61.9 ±11.8 | 0.417 |
| Sex (male) | 1291 (40.8) | 546 (41.5) | 0.709 |
| Hypertension | 1258 (39.8) | 471 (35.8) | 0.014 |
| Diabetes | 426 (13.5) | 205 (15.6) | 0.073 |
| Hyperlipidemia | 1062 (33.6) | 439 (33.3) | 0.908 |
| Current Smoking | 218 (6.9) | 88 (6.7) | 0.852 |
| Previous coronary artery disease | 417 (13.2) | 155 (11.8) | 0.215 |
| Previous stroke | 288 (9.1) | 125 (9.5) | 0.724 |
| Systolic blood pressure (mmHg) | 145.5 ±22.0 | 147.0 ±22.7 | 0.035 |
| Diastolic blood pressure (mmHg) | 87.9 ±14.1 | 88.5 ±14.3 | 0.149 |
| Heart rate (beat/min) | 74.5 ±14.1 | 75.0 ±14.3 | 0.297 |
| Revisit | 48 (1.5) | 7 (0.5) | 0.010 |

† Results represented as frequency (Percentage) or mean ± SD.

‡ *P*-values were calculated using t-test for continuous variables and χ2-test for categorical variables.

이 문서는 서울아산병원의 문서입니다. 동의없이 수정, 변경 및 복사할 수 없습니다.

**Supplementary Table 2.** Coefficients of logistic regression

| | Coef | SE | Z value | Pr(>\|z\|) |
|---|---|---|---|---|
| Sex | | | | |
| Female | reference | | | |
| Male | 0.85783 | 0.14095 | 6.086 | <0.001 |
| Age | 0.32322 | 0.07643 | 4.229 | <0.001 |
| Presentation | | | | |
| Vertigo | reference | | | |
| Dizziness | 0.94889 | 0.14704 | 6.453 | <0.001 |
| Hypertension | -0.01546 | 0.13777 | -0.112 | 0.911 |
| Diabetes | 0.53221 | 0.16574 | 3.211 | 0.001 |
| Current Smoking | 0.27698 | 0.22851 | 1.212 | 0.225 |
| Previous coronary artery disease | 0.1873 | 0.17241 | 1.086 | 0.277 |
| Previous stroke | 1.03002 | 0.1665 | 6.186 | <0.001 |
| Hyperlipidemia | 0.4384 | 0.14847 | 2.953 | 0.003 |
| Systolic blood pressure (mmHg) | 0.08749 | 0.08776 | 0.997 | 0.319 |
| Diastolic blood pressure (mmHg) | -0.05169 | 0.09112 | -0.567 | 0.571 |
| Heart rate (beat/min) | 0.05692 | 0.06548 | 0.869 | 0.385 |
| Intercept | -3.82293 | 0.17823 | -21.449 | <0.001 |

이 문서는 서울아산병원의 문서입니다. 동의없이 수정, 변경 및 복사할 수 없습니다.

**Supplementary Figure 1.** Relative feature importance of each feature

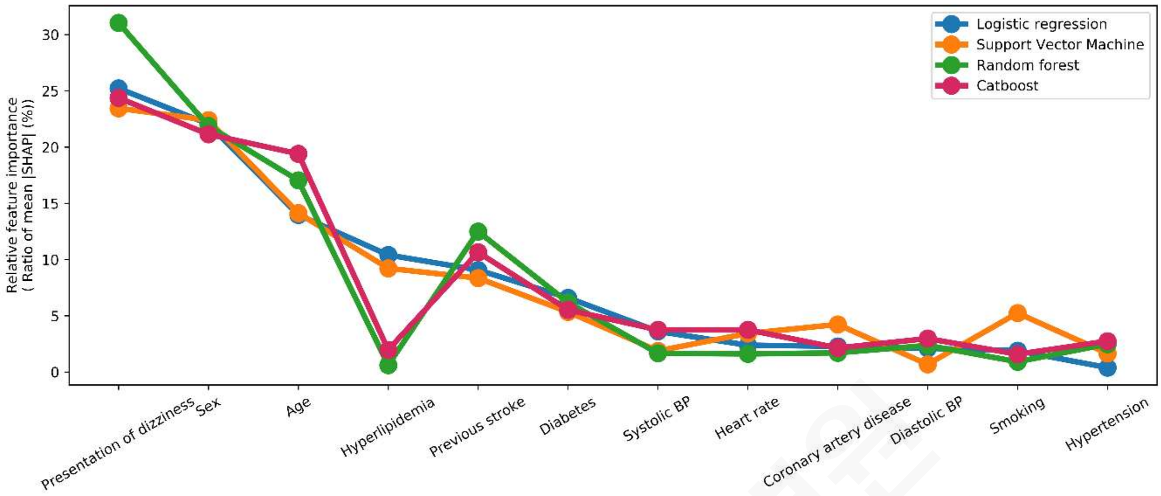


서울아산병원 Asan Medical Center
ASAN FOUNDATION 아산재단

이 문서는 서울아산병원의 문서입니다. 동의없이 수정, 변경 및 복사할 수 없습니다.

**Supplementary Figure 2.** Feature attributions for Support vector machine and Random forest.

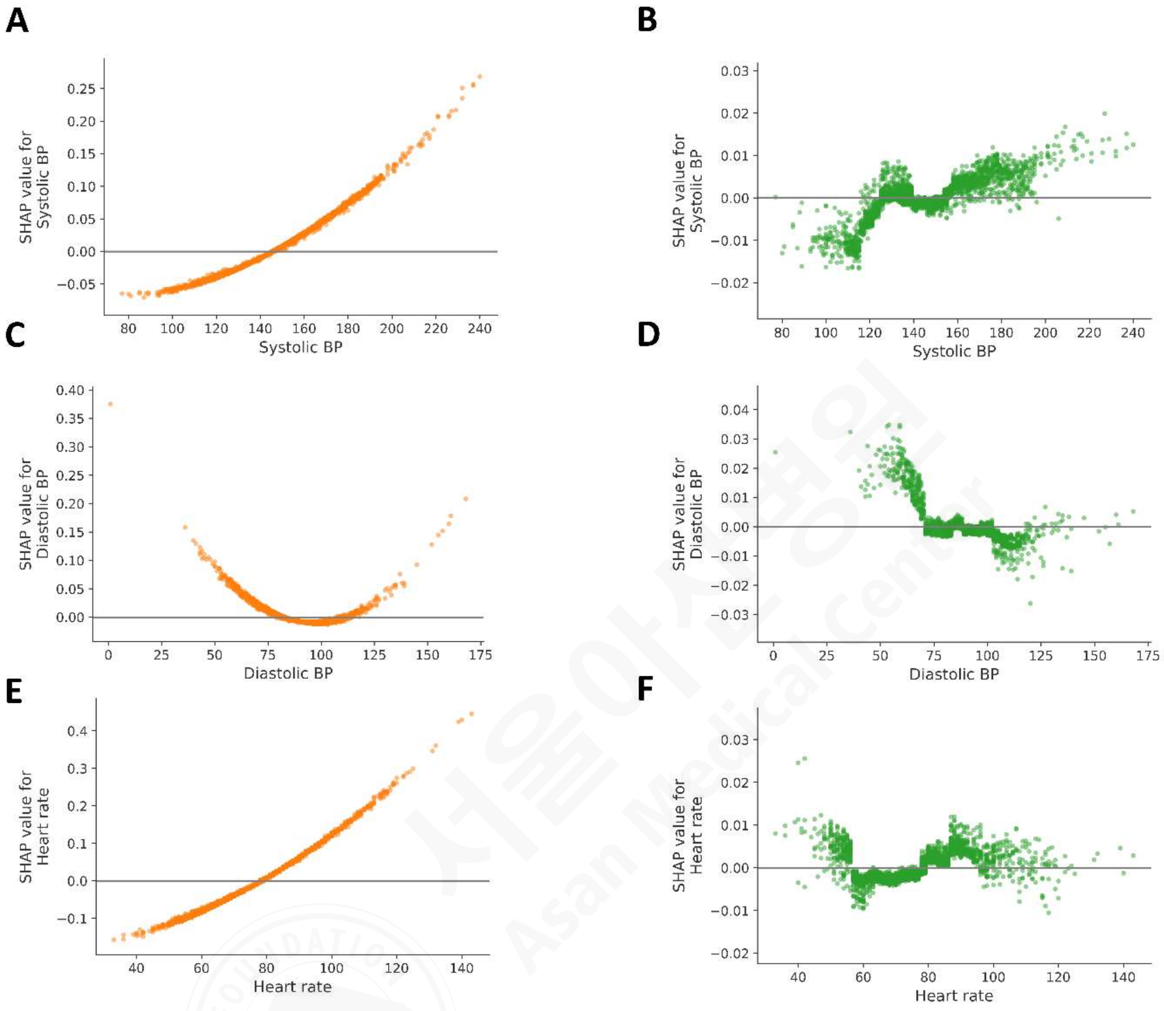

Supplement: Supplementary file 1 [file Data_Sheet_1.PDF]
